# Genome-scale mining of root-preferential genes from maize and characterization of their promoter activity

**DOI:** 10.1186/s12870-019-2198-8

**Published:** 2019-12-26

**Authors:** Ye Li, Xiaoqing Liu, Rumei Chen, Jian Tian, Yunliu Fan, Xiaojin Zhou

**Affiliations:** Biotechnology Research Institute, Chinese Academy of Agricultural Sciences, 12 ZhongGuanCun South Street, Beijing, 100081 China

**Keywords:** Maize, Promoter, Root, Root-preferential, *GUS*

## Abstract

**Background:**

Modification of root architecture and improvement of root resistance to stresses can increase crop productivity. Functional analyses of root-specific genes are necessary for root system improvement, and root-specific promoters enable research into the regulation of root development and genetic manipulation of root traits. Maize is an important crop species; however, little systematic mining of root-specific genes and promoters has been performed to date.

**Results:**

Genomic-scale mining based on microarray data sets followed by transcript detection resulted in the identification of 222 root-specific genes. Gene Ontology enrichment analyses revealed that these 222 root-specific genes were mainly involved in responses to chemical, biotic, and abiotic stresses. Of the 222 genes, 33 were verified by quantitative reverse transcription polymerase chain reaction, and 31 showed root-preferential activity. About 2 kb upstream 5 of the 31 identified root-preferential genes were cloned from the maize genome as putative promoters and named *p8463*, *p5023*, *p1534*, *p8531* and *p6629*. GUS staining of transgenic maize-derived *promoter-GUS* constructs revealed that the five promoters drove *GUS* expression in a root-preferential manner.

**Conclusions:**

We mined root-preferential genes and their promoters in maize and verified *p8463*, *p5023*, *p1534*, *p8531* and *p6629* as root-preferential promoters. Our research enables the identification of other tissue-specific genes and promoters in maize and other species. In addition, the five promoters may enable enhancement of target gene(s) of maize in a root-preferential manner to generate novel maize cultivars with resistance to water, fertilizer constraints, or biotic stresses.

## Background

The root is one of the most important organs in a plant, as it provides mechanical support; protects against abiotic stresses, including water deficits, low nutrients, and soil compaction; and protects against biotic stresses, such as pathogen infection. A plant’s root system can be modified to enhance the capture of water and nutrients and to sense and adapt to abiotic and biotic stresses, improving crop productivity [1–3]. The reverse genetics approach facilitates unraveling of gene functions. Thus, research into genes specifically expressed in roots is needed to understand root structure and function. Many root-specific genes have been functionally identified. For instance, the root-specific gene *DRO1* influences the root system architecture in *Arabidopsis* and *Prunus*, and *ZmLOX3* controls maize resistance to root-knot nematodes as a root-specific suppressor [4, 5]. Mining of root-specific promoters facilitates analyses of gene functions in root and further improves crop productivity [6, 7].

Appropriate temporal and spatial gene expression is crucial for the life cycle of all organisms. Promoters initiate and regulate gene transcription and play important roles in transgenic engineering. The use of a suitable promoter is a key determinant in plant genetic transformation. Constitutive promoters, such as the *35S* promoter and the maize *ubiquitin* promoter, are used to generate transgenic plants, although constitutive overexpression of an exogenous gene in unnecessary tissues typically has unexpected effects on growth and development and is a biosafety issue [8, 9]. Organ- or tissue-specific promoters enable adjustment of gene expression in a spatially controlled manner to avoid undesirable effects or excessive energetic costs to transgenic plants. Much effort has been devoted to identifying organ- or tissue-specific promoters, and many such promoters have been characterized and applied to genetic transformation of crops such as maize, rice, soybean, tomato, potato and tobacco [10–16].

Several root-specific promoters have been identified to date, such as *Pyk10* and *PHT1* from *Arabidopsis*, *ToRB7* and *NtREL1* from tobacco, *RCc3* from rice, *FaRB7* from strawberry, *SlREO* from tomato, and *SRD1* from sweet potato [17–24]. Most characterized root-specific promoters have been identified from dicotyledonous plants; few have been identified from monocotyledonous plants. Maize (*Zea mays L.*) is one of the most important food crops for both humans and livestock and is a model species for bioenergy research. Use of exogenous promoters to drive gene expression results in imperfect tissue specificity, which indicates a need to characterize native promoters for plant transformation [25–27]. Root-specific or -preferential promoters could enable the identification of essential pathways for root development and facilitate root enhancement in maize. However, to the best of our knowledge, few studies have characterized endogenous root-specific promoters in maize.

Microarray data allow for gene expression analyses in a variety of tissue or cell types and have been used to compare gene expression profiles, predict marker genes, and identify tissue- or cell-specific genes [28–32]. For instance, by analyzing gene expression in microarray data sets, researchers identified several rice root-specific promoters (*rRSP1–rRSP5*) and maize embryo-specific promoters (Zm.13387, Zm.85502, Zm.3896 and Zm.2941) [30, 31]. However, the screenings were not comprehensive because the data sets did not cover the whole life cycle of rice or maize, and candidate genes were not validated by RNA sequencing (RNAseq).

In this study, we used two strategies to identify root-preferential promoters. First, a large-scale analyses of two microarray data sets covering major developmental stages of maize tissues were performed to screen root-preferential genes. Second, the tissue specificity of candidate genes was verified by RNAseq and quantitative reverse transcription polymerase chain reaction (qRT-PCR). To ensure the reliability of this combined method and to characterize root-specific/−preferential promoters, we subjected five promoters of the candidate root-specific genes to GUS histochemical staining in stably transformed maize plants. This research is a useful resource for genetic manipulation of root traits, and the five characterized promoters have the potential to drive root-predominant gene expression in maize and other cereals or monocotyledonous plants.

## Results

### Screening of root-specific genes in maize

To identify root-specific genes, we performed genome-scale screening using a microarray data set consisting of 17,555 probe sets for 13,339 maize genes [30]. The expression of all probe sets in root relative to 13 non-root tissues (stem, stem tip, leaf, embryo, and endosperm at 10, 15, 20 and 25 days after pollination [DAP], ear, and silk) was compared with significance analysis of microarrays (SAM; Stanford University, Stanford, CA, USA) to identify significantly differentially expressed genes. Ten root-specific probe sets representing eight maize genes were filtered out (Additional file 1: Table S1).

**Fig. 1:**
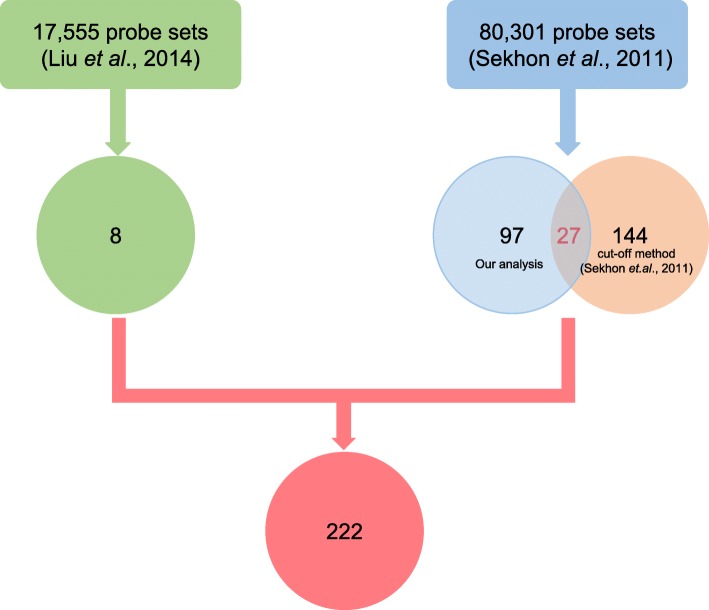
Schematic diagram of the strategy for identifying root-specific genes. Two microarray data sets containing 17,555 probe sets (green box) and 80,301 probe sets (blue box) were analyzed with SAM software, which yielded 8 and 97 root-specific probe sets, respectively. Together with 144 previously identified root-specific genes from 80,301 probe sets, 222 nonredundant candidate root-specific genes were identified

To identify more root-specific genes, we analyzed a comprehensive transcriptome data set containing 80,301 probe sets covering 60 maize tissues [33]. In these microarray data, roots were divided into three developmental stages: 6 days after sowing (DAS)_GH_primary root, vegetative emergence (VE)_primary root, and vegetative 1_GH_primary root. To identify root-specific genes, we compared the expression of each gene from roots at the three stages to that in the other 57 tissues by SAM. A total of 260 common differentially expressed probe sets were identified (Additional file 2: Table S2). Of them, 116 probe sets were mapped to official maize cDNA models and encoded by 97 high-confidence genes. Sekhon et al. used an expression cutoff method to identify 151 root-specific genes (actually 151 transcripts encoded by 144 genes). Thus, combining the 97 high-confidence genes in this study with the 144 previously identified root-specific genes, we obtained 214 root-specific probe sets (after removing duplicate genes) from 80,301 probe sets (Additional file 3: Table S3). Ultimately, a total of 222 root-specific genes from the above two probe sets (214 plus 8 genes) were identified (Fig. 1).

### GO enrichment analyses of root-specific genes

**Fig. 2:**
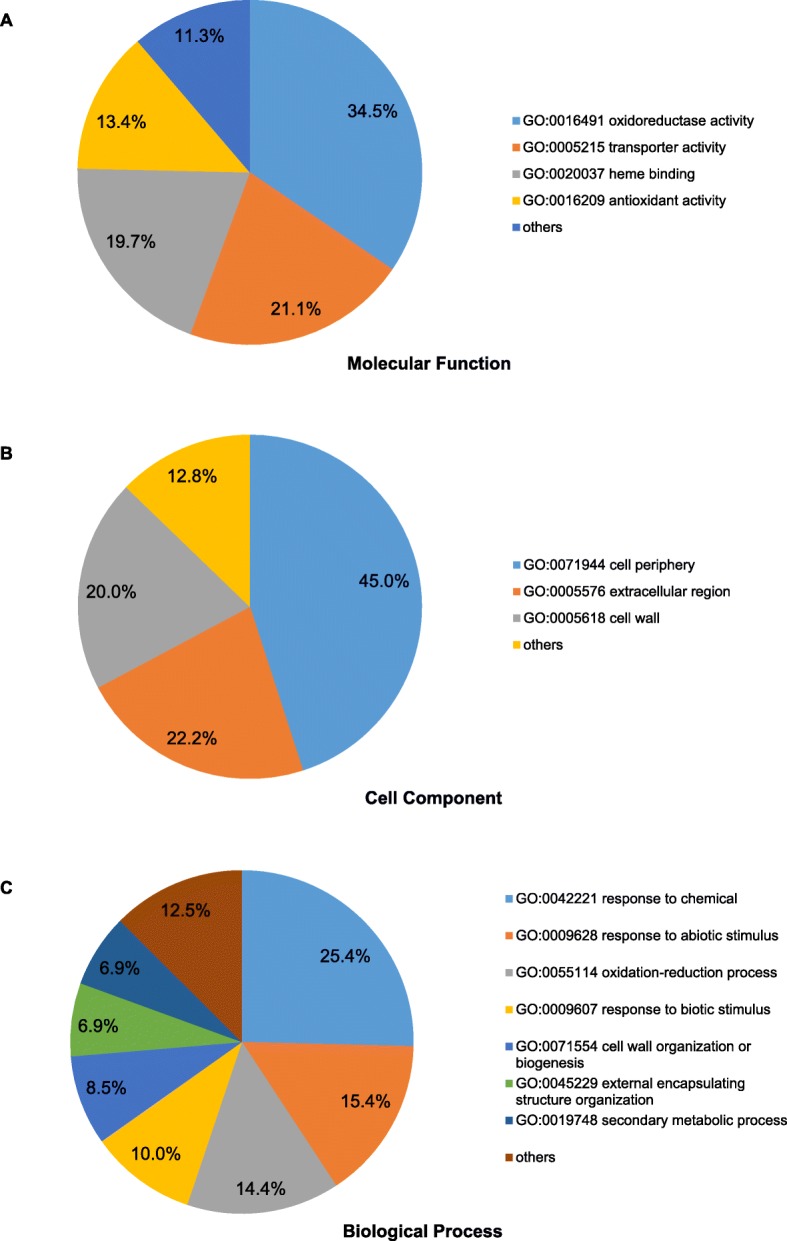
Gene Ontology (GO) classifications of root-specific genes. In total, 201 genes were annotated and classified with the agriGO Analyses Toolkit. Pie chart of significantly enriched terms in the molecular function (**a**), cellular component (**b**), and biological process (**c**) categories. Numbers are the proportions of genes in each category

Of the 222 root-specific genes, 201 were functionally annotated and classified with the GO database (http://bioinfo.cau.edu.cn/agriGO/). GO enrichment analyses of the 201 annotated genes identified 71 significantly (FDR < 0.05) enriched GO terms for the biological process (BP), molecular function (MF), and cellular component (CC) categories (Additional file 4: Table S4). Within the MF category (Fig. 2a), the largest proportion of the genes was enriched in oxidoreductase activity (GO 0016491; 34.5%), whereas transporter activity (GO 0005215) and heme binding (GO 0020037) accounted for 21.1 and 19.7%, respectively. In the CC category (Fig. 2b), cell periphery (GO 0071944) accounted for the highest proportion (45.0%), although extracellular region (GO 0005576; 22.2%) and cell wall (GO 0005618; 20.0%) were also enriched.

Within the BP category (Fig. 2c), response to chemicals (GO 0042221) was the most highly enriched (25.4%), including response to inorganic substances, oxygen-containing compounds, chemical stimuli, oxidative stress, and toxic substances. Response to abiotic stimulus (GO 0009628) and oxidation-reduction process (GO 0055114) were also enriched (15.4 and 14.4%, respectively), followed by response to biotic stimulus (GO 0009607; 10.0%), cell wall organization or biogenesis (GO 0071554; 8.5%), external encapsulating structure organization (GO 0045229; 6.9%), and secondary metabolic process (GO 0019748; 6.9%). Therefore, the majority of the 201 annotated candidate root-specific genes were involved in response to chemical, biotic, and abiotic stimuli, which suggests that genes that function in interactions with and adaptation to the soil environment tend to be expressed specifically in root.

### Validation of 33 candidate genes by RNAseq

A total of 27 high-confidence candidate genes were selected for further analyses, as they were screened in both our study (97 genes) and a previous study (144 genes) [33] (Fig. 1). Of the 27 candidate genes, 2 lacked sequence annotations (GRMZM2G168508 and GRMZM2G466823); therefore, 25 genes from 80,301 probe sets were selected. Combined with eight genes from 17,555 probe sets, 33 candidate root-specific genes were analyzed. Their functional features are listed in Additional file 5: Table S5.

**Fig. 3:**
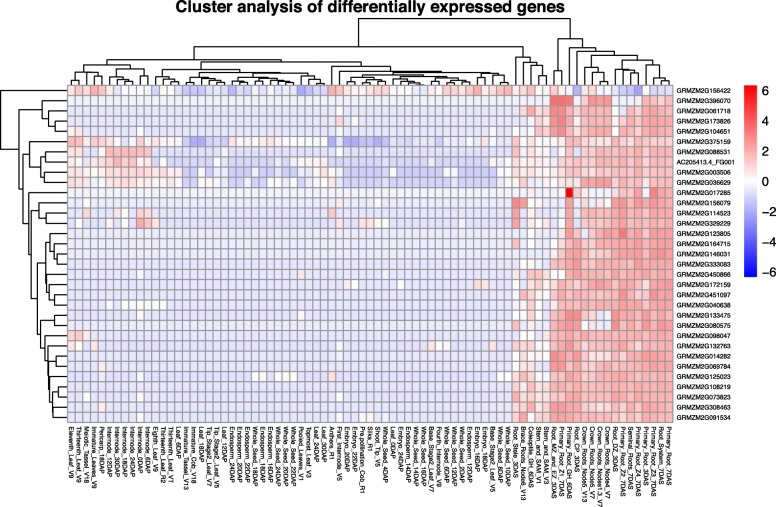
Heat map of the expression profiles of 33 root-preferential genes in 79 maize tissues. The heat map was generated with hierarchical clustering based on Pearson’s correlation. The RNAseq data comprised 79 maize samples, including vegetative (root, leaf, and stem) and reproductive (tassel, cob, endosperm, and embryo) tissues at different developmental stages. Scale bar, expression intensity as log_2_-fold change

To confirm the root specificity of the 33 genes, we analyzed their expression profiles using published information [34]. As shown in Fig. 3, all 33 genes were highly expressed in various root tissues. However, some were also highly expressed in other tissues; for example, GRMZM2G329229 was highly expressed in root as well as in 0 days after pollination (DAP) internode, whereas GRMZM2G156422 was highly expressed in not only root tissues but also leaf, internode, anther and seed. GRMZM2G375159 was highly expressed in both root and leaf. Thus, the expression profiles of the 33 candidate root-specific genes by RNAseq were generally consistent with those by microarray analyses, although there were some exceptions. In addition, some genes were highly expressed in non-root tissues, and so further analyses were needed.

### Expression of the 33 candidate genes by qRT-PCR

**Fig. 4:**
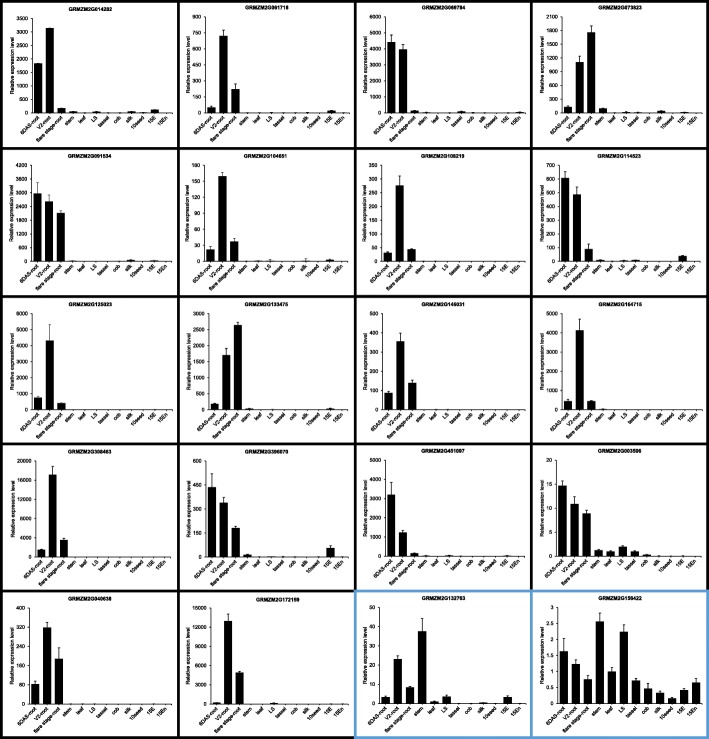
Expression of candidate genes with significant root specificity and low root preference. The expression of 18 genes (black borders) highly expressed in root at three stages was analyzed by qRT-PCR. Two candidate genes (blue borders) with low root preference are also shown. The maize *Actin1* gene was used as an internal control, and leaf was used as a reference sample. For qRT-PCR, three biological replicates were used, each with four technical replicates. Error bars indicate standard deviations

**Fig. 5:**
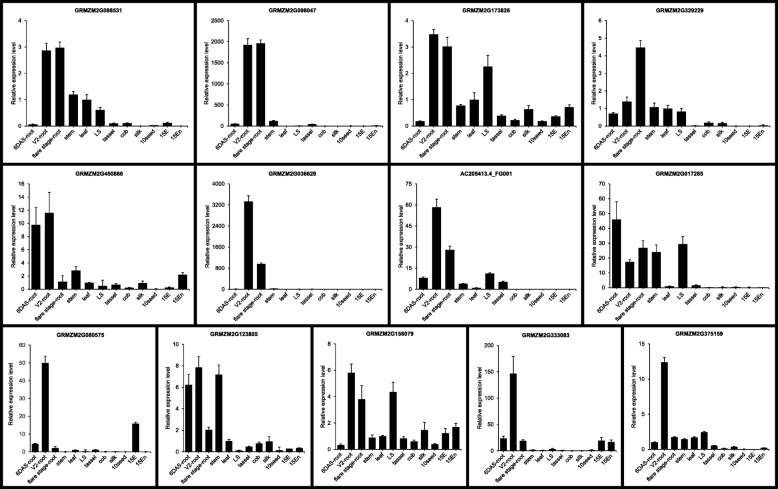
Expression of candidate genes with modest root-preferential expression. The expression of 13 genes highly expressed in one or two type(s) of root was analyzed by qRT-PCR. The expression of the maize *Actin1* gene was used as an internal control. 6 DAS-root was used as the calibrator for calculating the expression of GRMZM2G375159, whereas leaf was used as the reference sample for the other genes. Three biological replicates were used, each with four technical replicates. Error bars indicate standard deviations

To validate the tissue specificity of the 33 candidate root-specific genes, we examined their expression in 12 maize tissues by qRT-PCR. The 12 tissues were 6 DAS-root, vegetative 2 (V2)-root, flare stage-root, stem, leaf, leaf sheath (LS), tassel, cob, silk, 10 DAP seed (10 seed), 15 DAP embryo (15 E), and 15 DAP endosperm (15 En). As shown in Figs. 4, 18 genes displayed significant root specificity, as they were highly expressed in root at all three stages with low expression in other tissues. According to Figs. 5, 13 genes showed significantly higher expression in one or two type(s) of roots than any other tissue, which suggests modest root-preferential. However, GRMZM2G132763 was highly expressed in stem, and GRMZM2G156422 showed higher expression in stem and LS than in root of any stage (Fig. 4). Therefore, based on microarray and qRT-PCR data, we identified 31 root-preferential genes.

### Analyses of promoter activity in transgenic maize plants

To validate their root specificity, we selected five candidate promoters for transgenic analyses, including 3 (GRMZM2G308463, GRMZM2G125023 and GRMZM2G091534) of the 18 root-specific genes (Fig. 4) and 2 (GRMZM2G088531 and GRMZM2G036629) of the modest root-preferential genes. Approximately 2.0 kb upstream of ATG of these five root-preferential genes were cloned as putative promoter sequences and named *p8463*, *p5023*, *p1534*, *p8531* and *p6629* based on the last four digits of the gene ID. They were fused downstream with the *GUS* reporter gene in the *pCAMBIA3301* vector, which resulted in the constructs *p8463:GUS*, *p5023:GUS*, *p1534:GUS*, *p8531:GUS* and *p6629:GUS*. *Agrobacterium*-mediated transformation was used to generate transgenic maize plants harboring the *promoter-GUS* fusions. For individual constructs, we obtained at least four events (Additional file 6: Table S6).

**Fig. 6:**
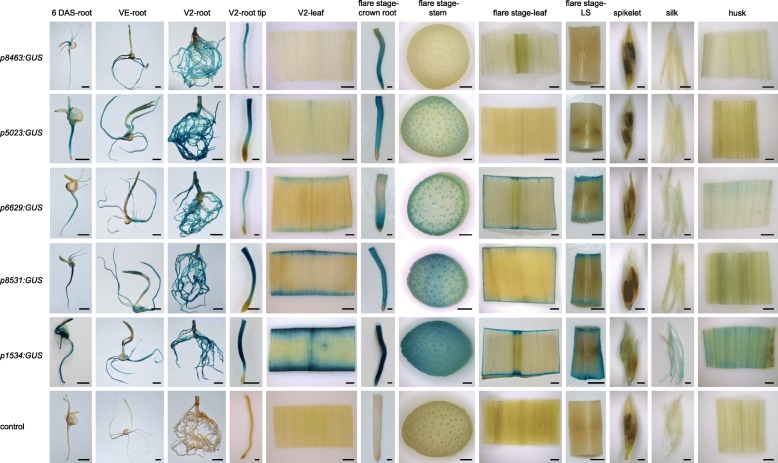
Validation of the tissue specificity of five putative root-preferential promoters in transgenic maize plants. Promoters of five root-preferential genes (GRMZM2G308463, GRMZM2G125023, GRMZM2G036629, GRMZM2G088531 and GRMZM2G091534) were analyzed for tissue specificity in transgenic maize plants. Approximately 2.0 kb upstream of the start codon were cloned as putative promoters and named *p8463*, *p5023*, *p1534*, *p8531* and *p6629*. Root tissues at four developmental stages and six non-root tissues of transgenic and wild-type (control) maize were subjected to GUS staining. For 6 DAS-root, VE-root, and V2-root, bar = 1 cm; for flare stage-LS, bar = 5 mm; for V2-root tip, bar = 0.5 mm; for V2-leaf, flare stage-crown root, flare stage-stem, flare stage-leaf, spikelet, silk, and husk, bar = 1 mm

To functionally characterize these five putative root-preferential promoters, we subjected root tissues at four developmental stages (6 DAS stage, VE, V2 stage and flare stage) as well as six non-root tissues (flare stage-stem, flare stage-leaf, flare stage-LS, spikelet, silk and husk) of transgenic maize and wild-type plants (control) to histochemical GUS staining. As shown in Figs. 6, 6 DAS-root, VE-root, V2-root and crown root of the five *promoter-GUS* transgenic maize plants at the flare stage exhibited strong staining, which indicates that the five putative promoters are active in root at all stages. In addition, the leaf, LS, spikelet, silk and husk of the *p5023:GUS* and *p8463:GUS* transgenic lines showed little GUS staining, and the stem of *p5023:GUS* showed slight staining. The *p6629:GUS*, *p8531:GUS* and *p1534:GUS* transgenic lines showed GUS staining in the cut-edge regions of V2-leaf, flare stage-stem, flare stage-leaf and flare stage-LS, which indicates that these promoters are induced by mechanical injury. Moreover, the *p1534:GUS* transgenic plants showed GUS staining in the spikelet, silk and husk, whereas *p6629:GUS* and *p8531:GUS* revealed little GUS staining in these tissues.

In summary, the five candidate promoters drove gene expression in root at all stages but exhibited different levels of tissue specificity. *p8463* was root specific, whereas *p5023* was highly root preferential. In addition, *p6629* and *p8531* were modestly root preferential, as they were inducible by wounding in stem, leaf and leaf sheath. *p1534* was also modestly root preferential, as it drove expression of *GUS* not only in root but also in stem, leaf, leaf sheath, spikelet, silk and husk in response to mechanical injury.

## Discussion

In this study, 222 genes with root-specific expression were identified by a combined method using two published microarray data sets. GO enrichment analyses demonstrated that the majority of the root-predominant genes functioned in response to a stimulus, consistent with the role of roots in protecting plants from the soil environment [35, 36]. The transcription of 33 selected high-confidence genes in different tissues was analyzed with an RNAseq database and their spatial and temporal expression were evaluated by qRT-PCR. Although 31 of the 33 genes showed root-preferential expression, their expression differed among three root developmental stages (Figs. 4 and 5); for example, transcription of GRMZM2G451097 in 6 DAS-root was about six-fold higher than in V2-root and about 61-fold higher than in flare stage-root. Transcription of GRMZM2G073823 increased with root development: the values were about 18 in 6 DAS-root, 131 in V2-root and 140 in flare stage-root. Therefore, the expression of some root-preferential genes in root differs according to developmental stage, which indicates that their functions vary with plant development.

Tissue-specific promoters have several advantages over constitutive promoters and thus are recommended for genetic manipulation. Root-specific promoters have numerous applications. Although several root-specific promoters have been characterized, these promoters do not drive target gene expression in an expected root-specific manner, such as reduced promoter activity and altered gene expression, in heterologous plants [20, 21, 37]. Hence, the endogenous root-specific promoters need to be mined and characterized.

The spatial and temporal expression of a gene depends on many factors, including the availability of transcription factors and *cis*-elements in promoters [38]. Analyses by PLACE and PlantCARE revealed TATABOX (TATAAAT/TATTAAT/TTATTT/TATTTAA) sequence elements, which play an important role in initiating transcription, in the five promoter sequences [39, 40]. CAATBOX (CAAT) elements, which contribute to tissue-specific promoter activity, were also found in the five promoter regions at numerous positions (Additional file 7: Table S7). ROOTMOTIFTAPOX1 (ATATT) are critical *cis*-elements required for root-specific expression, and OSE1ROOTNODULE (AAAGAT) and OSE2ROOTNODULE (CTCTT) elements are related to root-specific promoter activity [41–43]. The five promoters had ROOTMOTIFTAPOX1 and OSE2ROOTNODULE elements, and *p8531* and *p1534* also contained OSE1ROOTNODULE elements. Several other inducible elements were also found within the five promoter sequences, such as the wounding-inducible element WBOXNTERF3 (TGACY), the dehydration-inducible elements ABRELATERD1 (ACGTG) and ACGTATERD1 (ACGT), and the pathogen and salt stress-inducible element GT1GMSCAM4 (GAAAAA; Additional file 7: Table S7). LTRE1HVBLT49 is a low-temperature-responsive element of the *blt4.9* promoter in barley [44]. MYB2AT is a MYB recognition sequence that participates in the response to water stress in *Arabidopsis* [45]. These *cis*-elements were also found in the five promoter regions. In summary, *cis*-element prediction analyses suggested that ROOTMOTIFTAPOX1, OSE2ROOTNODULE, or OSE1ROOTNODULE may be responsible for the root-preferential expression driven by *p8463*, *p5023*, *p1534*, *p8531* and *p6629*; whereas other stress-inducible elements may lead to less strict root expression of *p5023*, *p1534*, *p8531* and *p6629*. In addition, uncharacterized root-specific *cis*-elements may contribute to root specificity. Therefore, further study is needed to analyze the mechanisms of root-preferential expression and determine whether other inducible *cis*-elements participate in regulating gene expression.

*p8463* was root specific, whereas *p5023* was highly root preferential. *ZmTIP2–3* (Gene ID: GRMZM2G125023) encodes a tonoplast intrinsic protein whose expression and function have been characterized [46]. Northern blot analyses revealed that *ZmTIP2–3* was specifically expressed in roots, which confirms our result that the transcript of *ZmTIP2–3* was root specific (Fig. 4). In addition, as homologues of *ZmTIP2–3*, *EgTIP2* from *Eucalyptus grandis* and *GmTIP2;3* from soybean show root-specific expression [24, 47]. This indicates that tonoplast intrinsic aquaporin genes are expressed specifically in root and that their promoters can drive root-preferential expression. GRMZM2G308463 encodes a NAD(P)-binding Rossmann-fold superfamily protein of unknown function. GUS staining showed that its promoter, *p8463*, can drive root-specific *GUS* expression (Figs. 4 and 6).

*p6629* and *p8531* were characterized as modestly root preferential, as they displayed low activity in stem, leaf and leaf sheath possibly induced by wounding. GRMZM2G036629 encodes metallothionein-like protein 1 (MT-L), which is a low molecular mass, metal-binding protein regulated by environmental stimuli, such as metal ions, wounding, heat shock and drought stress [48]. Our finding that GRMZM2G036629 was expressed predominantly in root and weakly in stem, leaf and seed (Fig. 5) is consistent with a previous report that the mRNA of MT-L was most abundant in root and less abundant in leaf, pith and kernel [49]. Maize GRMZM2G088531 encodes a polygalacturonase inhibitor. Polygalacturonase-inhibiting proteins are defense proteins with constitutive and/or tissue-specific expression, and most are induced by biotic stresses, such as fungi or insects, as well as mechanical wounding [50–55]. The promoter region of *p6629* and *p8531* contains a WBOXNTERF3 wounding-inducible element (Additional file 7: Table S7). Therefore, *GUS* expression in the cut edges of leaf, leaf sheath and stem of the *p6629:GUS* and *p8531:GUS* transgenic lines may be induced by wounding.

GRMZM2G091534 encodes an extensin-like protein, which functions in, for example, root hair growth, embryo development, and stress responses [24, 56–58]. The promoter sequence of *p1534* contained a variety of wounding-inducible, stress-responsive, and phytohormone-inducible elements, which may explain the strong GUS staining of the cut edges of stem, leaf and leaf sheath in *p1534:GUS*. Our results suggest that *p1534* is a good candidate not only for root-preferential expression but also for controlling wounding- or stress-inducible expression.

In this study, we characterized five promoters using transgenic maize plants; only *p8463* was strictly root specific. *p5023* was strongly root preferential, whereas *p6629*, *p8531* and *p1534* were modestly root preferential. Although these root-preferential promoters are less specific for gene expression in root, it provides more options for promoters with various levels of expression intensity and different tissue specificities. Therefore, we evaluated the strengths of the five promoters by comparing the expression of these genes in root to that of maize *ubiquitin1*. Transcription of GRMZM2G036629 was about 3.76-fold higher than that of *ubiquitin1*, whereas transcription of GRMZM2G125023, GRMZM2G308463, GRMZM2G088531 and GRMZM2G091534 was about 0.14-, 0.03-, 0.47-, and 0.06-fold lower, respectively (Additional file 9: Figure S1). Therefore, the activity of *p6629* may be stronger than that of the maize *ubiquitin1* promoter in root, and that of *p5023*, *p8463*, *p8531* and *p1534* is possibly weaker.

It is difficult to achieve fine regulation of transgene expression mainly because of the lack of available high-efficiency promoters. Here, we characterized five novel root-preferential promoters in maize. *p8463* was root specific, whereas *p5023* was highly root preferential, in maize. Moreover, *p6629, p8531* and *p1534* were root-preferential yet wounding-inducible promoters. Although *p5023*, *p6629*, p8531 and *p1534* were root preferential rather than root specific, they can be used to drive foreign gene expression in root to strengthen the root system, increase the uptake of water and nutrients, and improve resistance to pathogens.

## Conclusions

In this study, 222 maize root-specific genes were mined and subjected to GO analyses, and five root-preferential promoters were characterized by genome-scale expression screening, qRT-PCR, and activity analyses in transgenic plants. This enabled the development of strategies for generating root-specific genes and promoters that can be used to screen other tissue-specific genes in other species. The five identified root-preferential promoters show potential for maize bioengineering to improve root architecture, tolerance, and resistance to biotic and abiotic stresses.

## Methods

### Microarray data analyses and heat map

Microarray data sets were downloaded from https://www.ncbi.nlm.nih.gov/geo/query/acc.cgi. Genes differentially expressed in root and non-root tissues were analyzed by SAM, which enables mining of genes with significant tissue preferential expression from a set of microarray experiments developed at Stanford University Labs (https://statweb.stanford.edu/~tibs/SAM/). q < 0.001 was used to identify genes significantly differentially expressed in root. A heat map of the 33 root-preferential genes was generated with hierarchical clustering based on Pearson’s correlation.

### Plant materials and growth conditions

The maize inbred line B73 and the maize hybrid Hi-II were provided by the Maize Genetics Cooperation Stock Center (http://maizecoop.cropsci.uiuc.edu/stox-q.php). Wild-type B73, Hi-II, and transgenic maize plants were grown in a greenhouse under an 18 h light:6 h dark photoperiod with day and night temperatures of 22 °C and 28 °C, respectively. Root (6 DAS-root, V2-root, and flare stage-root), flare stage-stem, flare stage-leaf, flare stage-LS, tassel, cob, silk, 10 seed, 15 E, and 15 En tissues were collected from B73 plants and immediately placed on ice. The samples were frozen, ground into powder in liquid nitrogen, and stored at − 80 °C until use.

### RNA preparation and qRT-PCR

We extracted total RNA using TRIzol reagent (TaKaRa Bio, Shiga, Japan) following the manufacturer’s instructions. First-strand cDNA was synthesized by reactions with Oligo (dT) primer, TranScript RT/RI Enzyme Mix, TS reaction mix, and gDNA remover (TransGen Biotech, Beijing, China). To investigate spatial and temporal expression in maize tissues, we used the gene-specific primers listed in Additional file 8: Table S8. The maize *Actin1* gene was used as the internal control. qRT-PCR was performed with an ABI 7500 Real-Time PCR System (Applied Biosystems, Foster City, CA, USA). The PCR conditions were initial denaturation at 95 °C for 2 min, followed by 40 cycles of denaturation at 95 °C for 5 s and annealing/extension at 60 °C for 34 s. We analyzed data using ABI 7500 software (ver. 2.0.5) with the ΔΔC_T_ method [59]. For qRT-PCR analyses, three biological replicates were used, with four technical replicates per biological replicate.

### Promoter cloning and vector construction

About 2.0 kb upstream from ATG, the root-preferential genes GRMZM2G125023, GRMZM2G308463, GRMZM2G091534, GRMZM2G088531 and GRMZM2G036629 were cloned from genomic DNA of B73 maize and considered promoter sequences. To generate the *GUS* fusion constructs, we amplified the promoter fragments with restriction sites added to 5′- and 3′-ends of the primers (Additional file 8: Table S8). Next, the promoter sequences were cloned into the cloning vector *pEASY-Blunt* (TransGen Biotech) and confirmed by sequencing. Next, *p8463*, *p5023*, *p1534*, *p8531* and *p6629* were digested with *Sma*I/*Pst*I, *Hind*III/*Xba*I, *EcoR*I/*Xba*I, *Sma*I/*Xba*I, and *Sma*I/*Pst*I, respectively. The native *CaMV35S* promoter of the binary vector *pCAMBIA3301* was replaced with the digested promoter sequences by digestion and ligation. The final *GUS* fusion constructs were named *p8463:GUS*, *p5023:GUS*, *p1534:GUS*, *p8531:GUS* and *p6629:GUS* and were introduced into *Agrobacterium tumefaciens* strain EHA105.

### Generation of transgenic maize plants

*Agrobacterium*-mediated maize transformation was performed as described previously [60]. Briefly, immature embryos (1.0–2.0 mm diameter) were peeled off from ~ 10 DAP ears of Hi-II maize and immersed in liquid infection medium and prepared for infection. A single colony of *A. tumefaciens* strain EHA105 harboring *promoter-GUS* plasmids was incubated for 2 days, cultured on solid yeast extract-beef medium for 3 days, and scraped into liquid infection medium containing 1% acetosyringone and cultured until the optical density at OD_550_ reached 0.3–0.4. Then we infected the embryos and selected calli by adding 3 mg/L bialaphos to the medium. Transgenic T_0_ maize plants were painted with an herbicide for identification and were self-crossed to generate T_1_ seeds.

### GUS histochemical staining

Tissues from transgenic maize plants were subjected to histochemical staining for GUS activity. Samples were washed with water and incubated in GUS staining solution containing 50 mM phosphate buffer (pH 7.0), 0.1% (v/v) Triton X-100 (pH 7.0), 2 mM potassium ferrocyanide, 2 mM potassium ferricyanide, 10 mM ethylenediaminetetraacetic acid, and 2 mM 5-bromo-4-chloro-3-indolyl-b-D-glucuronide (X-Gluc) in darkness for 4–8 h at 37 °C. After histochemical staining, green-tissue samples were immersed in 95% ethanol for 1 or 2 days to remove chlorophyll. A Leica M165FC stereomicroscope (Leica, Wetzlar, Germany) was used to visualize GUS staining of the magnified V2-root tip, V2-leaf, flare stage-crown root, flare stage-stem, flare stage-leaf, flare stage-LS, spikelet, silk, and husk.

## Supplementary information

**Additional file 1: Table S1.** Ten root-specific probe sets filtered from the microarray data of Liu et al.

**Additional file 2: Table S2.** Two hundred sixty root-specific probe sets filtered from the microarray data of Sekhon et al.

**Additional file 3: Table S3.** Root-specific genes generated from the microarray data of Sekhon et al.

**Additional file 4: Table S4.** GO enrichment analyses of 222 root-specific genes.

**Additional file 5: Table S5.** Functional descriptions of the 33 candidate genes.

**Additional file 6: Table S6.** Transgenic events of the five *promoter-GUS* constructs.

**Additional file 7: Table S7.** Putative *cis*-acting elements in the region of the five promoters.

**Additional file 8: Table S8.** Primers used in this study.

**Additional file 9: Figure S1.** Relative expression of *ubiquitin1*, GRMZM2G125023, GRMZM2G308463, GRMZM2G036629, GRMZM2G088531 and GRMZM2G091534.

## Data Availability

All data and materials generated or analyzed during this study are included in this article or are available from the corresponding author on reasonable request.

## Abbreviations

BP: Biological process
CC: Cellular component
DAP: Days after pollination
DAS: Days after sowing
E: Embryo
En: Endosperm
GO: Gene ontology
LS: Leaf sheath
MF: Molecular function
MT-L: Metallothionein-like protein 1
qRT-PCR: Quantitative reverse transcription polymerase chain reaction
RNAseq: RNA sequencing
SAM: Significance analysis of microarrays
V2: Vegetative 2
VE: Vegetative emergence
X-Gluc: 5-bromo-4 chloro-3-indolyl-β-D- glucuronide
