# Supplementary material for: Genome-scale mining of root-preferential genes from maize and characterization of their promoter activity
Source: BMC Plant Biol. 2019 Dec 26;19:584. doi: 10.1186/s12870-019-2198-8 (PMC6933907; doi:10.1186/s12870-019-2198-8)
Supplement: Supplementary file 5 — Additional file 5: Table S5. Functional descriptions of the 33 candidate genes. [file 12870_2019_2198_MOESM5_ESM.docx]

**Additional file 5: Table S5.** Functional descriptions of the 33 candidate genes

| **Gene** | **Description** | **Gene function** |
| --- | --- | --- |
| GRMZM2G014282 | ABC transporter G family member 37 | ATP-dependent peptidase activity [[1](#_ENREF_1)] |
| GRMZM2G017285 | Cinnamoyl-CoA reductase 1 | Cinnamoyl-CoA reductase, involving in phenylpropanoid biosynthesis [[1](#_ENREF_1)] |
| GRMZM2G061718 | wall-associated receptor kinase 1 | protein serine/threonine kinase activity [[1](#_ENREF_1)] |
| GRMZM2G069784 | UPF0481 protein | unknown |
| GRMZM2G073823 | GRAS-transcription factor 68 | peptide-methionine (S)-S-oxide reductase activity [[1](#_ENREF_1)] |
| GRMZM2G080575 | Casparian strip membrane protein 5 | unknown |
| GRMZM2G088531 | Leucine-rich repeat (LRR) family protein | unknown |
| GRMZM2G091534 | extensin-like protein | unknown |
| GRMZM2G098047 | CASP-like protein 1U3 | unknown |
| GRMZM2G104651 | Probable xyloglucan endotransglucosylase/hydrolase protein 21 | hydrolase activity, hydrolyzing O-glycosyl compounds [[1](#_ENREF_1)] |
| GRMZM2G108219 | peroxidase14 | [Heme binding](https://www.ebi.ac.uk/QuickGO/term/GO:0020037), [metal ion binding](https://www.ebi.ac.uk/QuickGO/term/GO:0046872), [peroxidase activity](https://www.ebi.ac.uk/QuickGO/term/GO:0004601) [[1](#_ENREF_1)] |
| GRMZM2G114523 | Lysine histidine transporter-like 6 | rRNA processing [[1](#_ENREF_1)] |
| GRMZM2G123805 | Patatin-like protein 2 | lipid metabolic process [[1](#_ENREF_1)] |
| GRMZM2G125023 | aquaporin TIP2-3 | water transporter activity [[2](#_ENREF_2)] |
| GRMZM2G132763 | Leucine-rich repeat receptor-like protein kinase PEPR1 | protein serine/threonine kinase activity [[1](#_ENREF_1)] |
| GRMZM2G133475 | Peroxidase 66 | Involving in [hydrogen peroxide catabolic process](https://www.ebi.ac.uk/QuickGO/term/GO:0042744), [responsing to oxidative stress](https://www.ebi.ac.uk/QuickGO/term/GO:0006979) [[1](#_ENREF_1)] |
| GRMZM2G146031 | Cinnamoyl-CoA reductase 1 | 3-beta-hydroxy-delta5-steroid dehydrogenase activity [[1](#_ENREF_1)] |
| GRMZM2G156079 | ZCN2 | unknown |
| GRMZM2G164715 | TRICHOME BIREFRINGENCE-LIKE 20 | [unknown](http://www.pantherdb.org/panther/category.do?categoryAcc=GO:0016407) |
| GRMZM2G173826 | Expansin-A11 | [Plant-type cell wall organization](https://www.ebi.ac.uk/QuickGO/term/GO:0009664) [[1](#_ENREF_1)] |
| GRMZM2G308463 | NAD(P)-binding Rossmann-fold superfamily protein | alcohol dehydrogenase (NAD) activity [[1](#_ENREF_1)] |
| GRMZM2G329229 | Aluminum-activated malate transporter 10 | unknown |
| GRMZM2G333083 | G2-like-transcription factor 2 | homoserine kinase activity [[1](#_ENREF_1)] |
| GRMZM2G396070 | Cysteine proteinase inhibitor | unknown |
| GRMZM2G451097 | Peroxidase 64 | response to environmental stresses [[1](#_ENREF_1)] |
| GRMZM2G450866 | Ustilago maydis induced11 | Response to Ustilago maydis [[3](#_ENREF_3)] |
| GRMZM2G036629 | Metallothionein-like protein 1 | [Metal ion binding](https://www.ebi.ac.uk/QuickGO/term/GO:0046872) activity [[1](#_ENREF_1)] |
| GRMZM2G003506 | membrane protein precursor | unknown |
| GRMZM2G156422 | Lung seven transmembrane receptor family protein | integral component of membrane [[1](#_ENREF_1)] |
| GRMZM2G040638 | guaiacol peroxidase 2 | Hydrogen peroxide detoxification [[4](#_ENREF_4)] |
| GRMZM2G172159 | Uncharacterized protein | unknown |
| AC205413.4_FG001 | Peroxidase 64 | response to oxidative stress [[1](#_ENREF_1)] |
| GRMZM2G375159 | HXXXD-type acyl-transferase family protein | transferase activity, transferring acyl groups other than amino-acyl groups [[1](#_ENREF_1)] |

**References**

1. Jaiswal P, Avraham S, Ilic K, Kellogg EA, McCouch S, Pujar A, Reiser L, Rhee SY, Sachs MM, Schaeffer M *et al*: **Plant Ontology (PO): a controlled vocabulary of plant structures and growth stages.** *Comparative and Functional Genomics* 2005, **6**(7‐8):388-397.

2. Chaumont F, Barrieu F, Wojcik E, Chrispeels MJ, Jung R: **Aquaporins Constitute a Large and Highly Divergent Protein Family in Maize.** *Plant physiology* 2001, **125**(3):1206-1215.

3. Basse CW: **Dissecting defense-related and developmental transcriptional responses of maize during Ustilago maydis infection and subsequent tumor formation.** *Plant physiology* 2005, **138**(3):1774-1784.

4. de Obeso M, Caparros-Ruiz D, Vignols F, Puigdomenech P, Rigau J: **Characterisation of maize peroxidases having differential patterns of mRNA accumulation in relation to lignifying tissues.** *Gene* 2003, **309**(1):23-33.
