# Supplementary material for: Genome-scale mining of root-preferential genes from maize and characterization of their promoter activity
Source: BMC Plant Biol. 2019 Dec 26;19:584. doi: 10.1186/s12870-019-2198-8 (PMC6933907; doi:10.1186/s12870-019-2198-8)
Supplement: Supplementary file 6 — Additional file 6: Table S6. Transgenic events of the five promoter-GUS constructs. [file 12870_2019_2198_MOESM6_ESM.docx]

**Additional file 6: Table S6.** Transgenic events of five *promoter-GUS* constructs

| **Construct name** | **events** |
| --- | --- |
| *p5023:GUS* | 7 |
| *p8463:GUS* | 12 |
| *p6629:GUS* | 4 |
| *p8531:GUS* | 14 |
| *p1534:GUS* | 4 |
