# Supplementary material for: Genome-scale mining of root-preferential genes from maize and characterization of their promoter activity
Source: BMC Plant Biol. 2019 Dec 26;19:584. doi: 10.1186/s12870-019-2198-8 (PMC6933907; doi:10.1186/s12870-019-2198-8)
Supplement: Supplementary file 7 — Additional file 7: Table S7. Putative cis-acting elements in the region of the five promoters. [file 12870_2019_2198_MOESM7_ESM.docx]

**Additional file 7: Table S7.** Putative *cis*-acting elements in the region of the five promoters

**Table S7a** Putative *cis*-acting elements in the region of the *p5023*

| **Name of *cis*-element** | **Sequence** | **Element**  **number** | **Position from translation start site** | **Function** |
| --- | --- | --- | --- | --- |
| **Essential elements** |  |  |  |  |
| TATABOX | TATAAAT,  TATTAAT,  TTATTT, TATTTAA | 6 | +1872, -1575, +1483, +1091, -927, -706 | Essential for accurate transcription initiation [[1](#_ENREF_1)] |
| CAATBOX | CAAT | 22 | -1830, -1811, +1795, +1727, -1612, +1586, -1477, +1228, +1149, +1148, -1139, -1065, +1063, +1006, -902, -860, -756, -744, -347, +331, -327, -295 | Related to tissue-preferred promoter activity [[2](#_ENREF_2)] |
| **Root-specific elements** |  |  |  |  |
| OSE2ROOTNODULE | CTCTT | 6 | -1628, +1529, +1289, -1217, -508, -444 | Organ-specific element especially in infected cells of root nodules [[3-5](#_ENREF_3)] |
| ROOTMOTIFTAPOX1 | ATATT | 7 | -1202, +1090, +830, -829, +713, -712, +668 | Root specific element [[6](#_ENREF_6)] |
| **Other elements** |  |  |  |  |
| WBOXNTERF3 | TGACY | 4 | -1256, -448, +106, +40 | Involved in response to wounding [[7](#_ENREF_7)] |
| ABRELATERD1 | ACGTG | 4 | +1867, +1689, -1395, -674 | Involved in response to dehydration and etiolation [[8](#_ENREF_8)] |
| ACGTATERD1 | ACGT | 14 | +/-1867, +/-1689, +/-1430, +/-1396, +/-856, +/-675, +/-484 | Involved in response to dehydration [[8](#_ENREF_8)] |
| GT1GMSCAM4 | GAAAAA | 3 | +1993, -1409, +724 | Involved in response to pathogen and salt stress [[9](#_ENREF_9)] |

**Table S7b** Putative *cis*-acting elements in the region of *p8463*

| **Name of *cis*-element** | **Sequence** | **Element**  **number** | **Position from translation start site** | **Function** |
| --- | --- | --- | --- | --- |
| **Essential elements** |  |  |  |  |
| TATABOX | TATAAAT,  TATTAAT,  TTATTT, TATTTAA | 19 | +1218, +1217, +1149, -1148, +1127, +1126, -1109, +1099, +1098, +724, +472, -341, +266, -265, -221, -219, +206, -205, +179 | Essential for accurate transcription initiation [[1](#_ENREF_1)] |
| CAATBOX | CAAT | 21 | -1955, -1909, +1525, +1524, +1507, +1503, -1485, -1376, +1064, +1044, -977, +975, -848, +737, +719, +712, +711, +664, +663, -514, +333 | Related to tissue-preferred promoter activity [[2](#_ENREF_2)] |
| **Root-specific elements** |  |  |  |  |
| OSE2ROOTNODULE | CTCTT | 4 | +984, -890, -547, +20 | Organ-specific element especially in infected cells of root nodules [[3-5](#_ENREF_3)] |
| ROOTMOTIFTAPOX1 | ATATT | 13 | +1992, +1195, +1182, -1113, +1058, +1003, +721, -720, -525, -374, -359, -299, +64 | Root specific element [[6](#_ENREF_6)] |
| **Other elements** |  |  |  |  |
| WBOXNTERF3 | TGACY | 9 | +1746, -1369, -1316, -1273, +740, +390, -285, +164, +136 | Involved in response to wounding [[7](#_ENREF_7)] |
| ABRELATERD1 | ACGTG | 1 | 1437 | Involved in response to dehydration and etiolation [[8](#_ENREF_8)] |
| ACGTATERD1 | ACGT | 12 | +/-1438, +/-1235, +/-1089, +/-1024, +/-902, +/-324 | Involved in response to dehydration [[8](#_ENREF_8)] |
| GT1GMSCAM4 | GAAAAA | 1 | 1006 | Involved in response to pathogen and salt stress [[9](#_ENREF_9)] |

**Table S7c** Putative *cis*-acting elements in the region of *p6629*

| **Name of *cis*-element** | **Sequence** | **Element**  **number** | **Position from translation start site** | **Function** |
| --- | --- | --- | --- | --- |
| **Essential elements** |  |  |  |  |
| TATABOX | TATAAAT,  TATTAAT,  TTATTT, TATTTAA | 13 | +1558, -1467, -1460, -1446, -1304, +1303, +821, +702, +701, -601, -548, +246, +45 | Essential for accurate transcription initiation [[1](#_ENREF_1)] |
| CAATBOX | CAAT | 10 | +1837, +1836, +1765, -1404, -1262, +1179, +965, -755, +279, -217 | Related to tissue-preferred promoter activity [[2](#_ENREF_2)] |
| **Root-specific elements** |  |  |  |  |
| OSE2ROOTNODULE | CTCTT | 8 | -1900, -1497, -1243, +921, -886, -859, +769, +93 | Organ-specific element especially in infected cells of root nodules [[3-5](#_ENREF_3)] |
| ROOTMOTIFTAPOX1 | ATATT | 8 | +1299, -1296, -1180, -819, +753, -638, +552, -551 | Root specific element [[6](#_ENREF_6)] |
| **Other elements** |  |  |  |  |
| WBOXNTERF3 | TGACY | 1 | +1778 | Involved in response to wounding [[7](#_ENREF_7)] |
| WBOXATNPR1 | TTGAC | 5 | -1491, -1484, +1405, -1265, +979 | Involved in salicylic acid and pathogen induction [[10](#_ENREF_10)] |
| ABRELATERD1 | ACGTG | 2 | -1654, -68 | Involved in response to dehydration and etiolation [[8](#_ENREF_8)] |
| ACGTATERD1 | ACGT | 10 | +/-1655, +/-1374, +/-1162, +/-447, +/-69 | Involved in response to dehydration [[8](#_ENREF_8)] |
| GT1GMSCAM4 | GAAAAA | 6 | -1666, +1039, -1011, +478, +196, -35 | Involved in response to pathogen and salt stress [[9](#_ENREF_9)] |
| MYB2AT | TAACTG | 1 | +791 | Involved in drought response [[11](#_ENREF_11)] |

**Table S7d** Putative *cis*-acting elements in the region of *p8531*

| Name of *cis*-element | Sequence | Element  number | Position from translation start site | Function |
| --- | --- | --- | --- | --- |
| **Essential elements** |  |  |  |  |
| TATABOX | TATAAAT,  TTATTT | 5 | +1867, -1406, +354, -176, -149 | Essential for accurate transcription initiation [[1](#_ENREF_1)] |
| CAATBOX | CAAT | 28 | -1976, +1701, +1628, -1484, +1347, +1204, +1149, -1137, +1120, +1119, +978, +961, +960, +951, +928, -911, -901, +831, +653, +652, +585, +534, +542, -514, +426, +425, -397, -140 | Related to tissue-preferred promoter activity [[2](#_ENREF_2)] |
| **Root-specific elements** |  |  |  |  |
| OSE1ROOTNODULE | AAAGAT | 2 | +789, +208 | Organ-specific element especially in infected cells of root nodules [[3-5](#_ENREF_3)] |
| OSE2ROOTNODULE | CTCTT | 4 | -1004, +862, -496, +299 | Organ-specific element especially in infected cells of root nodules [[3-5](#_ENREF_3)] |
| ROOTMOTIFTAPOX1 | ATATT | 14 | -1894, -1871, -1590, -1205, -973, -627, -427, +395, -386, +348, -347, +289, -286, +180 | Root specific element [[12](#_ENREF_12), [13](#_ENREF_13)] |
| **Other elements** |  |  |  |  |
| WBOXNTERF3 | TGACY | 5 | +1666, +1280, -936, -747, -550 | Involved in response to wounding [[7](#_ENREF_7)] |
| WBOXATNPR1 | TTGAC | 2 | -937, -748 | Involved in salicylic acid and pathogen induction [[10](#_ENREF_10)] |
| LTRE1HVBLT49 | CCGAAA | 4 | -1122, -980, +454, +433 | Involved in response to low-temperature [[14](#_ENREF_14)] |
| ACGTATERD1 | ACGT | 6 | +/-1470, +/-1128, +/-605 | Involved in response to dehydration [[8](#_ENREF_8)] |
| GT1GMSCAM4 | GAAAAA | 3 | +1585, +713, -411 | Involved in response to pathogen and salt stress [[11](#_ENREF_11)] |

**Table S7e** Putative *cis*-acting elements in the region of *p1534*

| Name of *cis*-element | Sequence | Element  number | Position from translation start site | Function |
| --- | --- | --- | --- | --- |
| **Essential elements** |  |  |  |  |
| TATABOX | TATAAAT,  TTATTT, TATTTAA | 22 | +1863, +1862, +1651, +1415, +1393, -1358, -1341, +1250, -1226, -965, -961, -927, +921, +920, -862, +722, +230, -225, +32, -31, -29, +22 | Essential for accurate transcription initiation [[1](#_ENREF_1)] |
| CAATBOX | CAAT | 25 | +1996, +1786, +1742, +1502, +1501, -1486, +1484, -1472, -1442, -1363, -1347, +1336, +1335, +1303, -1256, -1195, -1154, -1148, -1142, -938, +699, -676, -647, -113 | Related to tissue-preferred promoter activity [[2](#_ENREF_2)] |
| **Root-specific elements** |  |  |  |  |
| ROOTMOTIFTAPOX1 | ATATT | 15 | +1440, 1345, -1344, +892, -891, +866, -865, +827, +792, +763, +229, -228, +153, +111, -42 | Root specific element [[6](#_ENREF_6)] |
| OSE1ROOTNODULE | AAAGAT | 2 | +835, +841 | Organ-specific element especially in infected cells of root nodules [[3-5](#_ENREF_3)] |
| OSE2ROOTNODULE | CTCTT | 3 | -1537, -1406, +78 | Organ-specific element especially in infected cells of root nodules [[3-5](#_ENREF_3)] |
| **Other elements** |  |  |  |  |
| WBOXNTERF3 | TGACY | 4 | -1971, -1071, -1047, -996 | Involved in response to wounding [[7](#_ENREF_7)] |
| WBOXATNPR1 | TTGAC | 3 | -1972, -1603, -1072 | Involved in salicylic acid and pathogen induction [[10](#_ENREF_10)] |
| ABRELATERD1 | ACGTG | 2 | -462, -873 | Involved in response to dehydration and etiolation [[8](#_ENREF_8)] |
| ACGTATERD1 | ACGT | 6 | +/-1497, +/-874, +/-463 | Involved in response to dehydration [[8](#_ENREF_8)] |
| GT1GMSCAM4 | GAAAAA | 4 | +1513, +422, +671, +711 | Involved in response to pathogen and salt stress [[9](#_ENREF_9)] |
| LTRE1HVBLT49 | CCGAAA | 4 | -1122, -980, +454, +433 | Involved in response to low-temperature [[14](#_ENREF_14)] |
| MYB2AT | TAACTG | 1 | +807 | Involved in drought response [[11](#_ENREF_11)] |

Y = T or C

**References**

1. Grace ML, Chandrasekharan MB, Hall TC, Crowe AJ: Sequence and spacing of TATA box elements are critical for accurate initiation from the beta-phaseolin promoter. J Biol Chem. 2004; 279(9):8102-8110.

2. Shirsat A, Wilford N, Croy R, Boulter D: Sequences responsible for the tissue specific promoter activity of a pea legumin gene in tobacco. Mol Gen Genet. 1989; 215(2):326-331.

3. Stougaard J, Petersen TE, Marcker KA: Expression of a complete soybean leghemoglobin gene in root nodules of transgenic Lotus corniculatus. Proc Natl Acad Sci U S A. 1987; 84(16):5754-5757.

4. Vieweg MF, Fruhling M, Quandt HJ, Heim U, Baumlein H, Puhler A, Kuster H, Andreas MP: The promoter of the Vicia faba L. leghemoglobin gene VfLb29 is specifically activated in the infected cells of root nodules and in the arbuscule-containing cells of mycorrhizal roots from different legume and nonlegume plants. Mol Plant Microbe In. 2004; 17(1):62-69.

5. Fehlberg V, Vieweg MF, Dohmann EM, Hohnjec N, Puhler A, Perlick AM, Kuster H: The promoter of the leghaemoglobin gene VfLb29: functional analysis and identification of modules necessary for its activation in the infected cells of root nodules and in the arbuscule-containing cells of mycorrhizal roots. J Exp Bot. 2005; 56(413):799-806.

6. Elmayan T, Tepfer M: Evaluation in tobacco of the organ specificity and strength of the rolD promoter, domain A of the 35S promoter and the 35S2 promoter. Transgenic Res. 1995; 4(6):388-396.

7. Nishiuchi T, Shinshi H, Suzuki K: Rapid and transient activation of transcription of the ERF3 gene by wounding in tobacco leaves: possible involvement of NtWRKYs and autorepression. J Biol Chem. 2004; 279(53):55355-55361.

8. Simpson SD, Nakashima K, Narusaka Y, Seki M, Shinozaki K, Yamaguchi-Shinozaki K: Two different novel cis-acting elements of erd1, a clpA homologous Arabidopsis gene function in induction by dehydration stress and dark-induced senescence. Plant J. 2003; 33(2):259-270.

9. Park HC, Kim ML, Kang YH, Jeon JM, Yoo JH, Kim MC, Park CY, Jeong JC, Moon BC, Lee JH *et al*: Pathogen- and NaCl-induced expression of the SCaM-4 promoter is mediated in part by a GT-1 box that interacts with a GT-1-like transcription factor. Plant Physiol. 2004; 135(4):2150-2161.

10. Yu D, Chen C, Chen Z: Evidence for an Important Role of WRKY DNA Binding Proteins in the Regulation of <em>NPR1</em> Gene Expression. Plant Cell. 2001; 13(7):1527-1540.

11. Urao T, Yamaguchi-Shinozaki K, Urao S, Shinozaki K: An Arabidopsis myb homolog is induced by dehydration stress and its gene product binds to the conserved MYB recognition sequence. Plant Cell. 1993; 5(11):1529-1539.

12. Benfey PN, Ren L Fau - Chua NH, Chua NH: The CaMV 35S enhancer contains at least two domains which can confer different developmental and tissue-specific expression patterns. (0261-4189 (Print)).

13. Keller B, Baumgartner C: Vascular-specific expression of the bean GRP 1.8 gene is negatively regulated. Plant Cell. 1991; 3(10):1051-1061.

14. Dunn MA, White AJ, Vural S, Hughes MA: Identification of promoter elements in a low-temperature-responsive gene (blt4.9) from barley (Hordeum vulgare L.). Plant Mol Biol. 1998; 38(4):551-564.
