# Supplementary material for: Genome-scale mining of root-preferential genes from maize and characterization of their promoter activity
Source: BMC Plant Biol. 2019 Dec 26;19:584. doi: 10.1186/s12870-019-2198-8 (PMC6933907; doi:10.1186/s12870-019-2198-8)
Supplement: Supplementary file 8 — Additional file 8: Table S8. Primers used in this study. [file 12870_2019_2198_MOESM8_ESM.docx]

**Additional file 8: Table S8.** Primers used in this study

Gene specific primers used for qRT-PCR

| **Gene** | **Primer** | **Sequences** | **Product length (bp)** |
| --- | --- | --- | --- |
| GRMZM2G014282 | RT4282F | 5’-GTTCTAGCAGCCTTCCCAATAT-3’ | 122 |
|  | RT4282R | 5’-GTTGCACTATGCAAGCACTAT-3’ |  |
| GRMZM2G017285 | RT7285F | 5’-GAGAATGGTCATATCCCAGTA-3’ | 119 |
|  | RT7285R | 5’-ATACAAAGTCCAACTTTACCA-3’ |  |
| GRMZM2G061718 | RT1718F | 5’-GGAAGAGGAAATGTTGTTGTCAGC-3’ | 122 |
|  | RT1718R | 5’-GTCCTTTGGTTTGATACATGC-3’ |  |
| GRMZM2G069784 | RT9784F | 5’-GCACTCCTGTCTATCAAAAAGT-3’ | 138 |
|  | RT9784R | 5’-AAAATACGCAAGCAACGTCATT-3’ |  |
| GRMZM2G073823 | RT3823F | 5’-GCCTATCCACATGGGTTGCACAA-3’ | 176 |
|  | RT3823R | 5’-TGGTGACAGAGATTTTAGGCCCC-3’ |  |
| GRMZM2G080575 | RT0575F | 5’-GGTCCTGCTCATAGTCCTCATC-3’ | 115 |
|  | RT0575R | 5’-TTGGTCTTCACTGCGGTCATG-3’ |  |
| GRMZM2G088531 | RT8531F | 5’-ACAGCTACCAGCACAACAAG-3’ | 142 |
|  | RT8531R | 5’-TACTTTACCTTGCTGGAACG-3’ |  |
| GRMZM2G091534 | RT1534F | 5’-TAGAGCCGGCCGTTTTGATGA-3’ | 139 |
|  | RT1534R | 5’-ATTCATCAAGCAGATTGCCGG-3’ |  |
| GRMZM2G098047 | RT8047F | 5'-GTGCATATCTCCGTCTACCTCGC-3' | 158 |
|  | RT8047R | 5'-TAATGCTTGTGGCGGCAGCTATGG-3' |  |
| GRMZM2G104651 | RT4651F | 5’-ACGACCTCGAGATAATGTGGGG-3’ | 110 |
|  | RT4651R | 5’-AGTTCCCAGGGACGAGCTTGAT-3’ |  |
| GRMZM2G108219 | RT8219F | 5’-GCCGACGCGTGAAATGATGAT-3’ | 87 |
|  | RT8219R | 5’-CCGATGATGCAGCAAGCCTTA-3’ |  |
| GRMZM2G114523 | RT4523F | 5’-GCATGTACCTCTACACTCACAT-3’ | 151 |
|  | RT4523R | 5’-AGTGTACAGTTTGCTGCATCGT-3’ |  |
| GRMZM2G123805 | RT3805F | 5’-TCATATTGCACTGTTTCAACA-3’ | 157 |
|  | RT3805R | 5’-GGATGGGTGTAGTTTTAATA-3’ |  |
| GRMZM2G125023 | RT5023F | 5’-CCAGCAGGAGTACCCATGAAAG-3’ | 190 |
|  | RT5023R | 5’-TCTCGCTTACGGCATCTTGG-3’ |  |
| GRMZM2G132763 | RT2763F | 5’-TTGATGAGGATAGATGATCAGT-3’ | 121 |
|  | RT2763R | 5’-TGATAACAACCAGAACACCT-3’ |  |
| GRMZM2G133475 | RT3475F | 5’-TGCTGGAAGGTCAACTCGTAA-3’ | 150 |
|  | RT3475R | 5’-CGTACACTTACTCGACCAACACC-3’ |  |
| GRMZM2G146031 | RT6031F | 5’-AGTGGTCAGCCTACAGGAGAAT-3’ | 116 |
|  | RT6031R | 5’-CCAACTGAGGCATTAACACC-3’ |  |
| GRMZM2G156079 | RT6079F | 5’-ACGCTAACCGTACGGCTCAA-3’ | 149 |
|  | RT6079R | 5’-GCCAAGCAGCTGATTAG-3’ |  |
| GRMZM2G164715 | RT4715F | 5’-GGAACGACATGCTGCTTCAT-3’ | 102 |
|  | RT4715R | 5’-GAGGGAGCACCATATTGTACAC-3’ |  |
| GRMZM2G173826 | RT3826F | 5’-CCAGACCTTCACCAACGGCAAT-3’ | 118 |
|  | RT3826R | 5’-GAAGGTGAAAGCAAATCGTCGGGC-3’ |  |
| GRMZM2G308463 | RT8463F | 5’-GGCAAGAACATCAATGGC-3’ | 118 |
|  | RT8463R | 5’-AAGTCGCCCAGGGTGTGTGAAGTT-3’ |  |
| GRMZM2G329229 | RT9229F | 5’-GAGATGACGATCAAAGTGCAC-3’ | 151 |
|  | RT9229R | 5’-TCGAGTTCGTCGTGTCTGATC-3’ |  |
| GRMZM2G333083 | RT3083F | 5’-AGGATGCCAGGAGGTTAGA-3’ | 127 |
|  | RT3083R | 5’-CGGCCATGAAACTTATCTCC-3’ |  |
| GRMZM2G396070 | RT6070F | 5’-AACAACTACTGGATGGTGCT-3’ | 135 |
|  | RT6070R | 5’-TCACATGGTGCCATTGAAGGT-3’ |  |
| GRMZM2G451097 | RT1097F | 5’-GAATCAAGACCAGCACCGAC-3’ | 108 |
|  | RT1097R | 5’-CATGCTATTGACCATGGCTT-3’ |  |
| GRMZM2G450866 | RT0866F | 5’-TCAGGAAAGGCGTCGTGCT-3’ | 144 |
|  | RT0866R | 5’-AGCTCACCACCCCGTGCAAT-3’ |  |
| GRMZM2G036629 | RT6629F | 5’-AGACGAAGAAGGCGGCGCAGTT-3’ | 147 |
|  | RT6629R | 5’-AGGTGACATCCATGCATGCCGC-3’ |  |
| GRMZM2G003506 | RT3506F | 5’-GATCGTTGCTGATGTGTTTC-3’ | 135 |
|  | RT3506R | 5’-ACAAATGGCACTATAGAACCC-3’ |  |
| GRMZM2G156422 | RT6422F | 5’-GTACCTGTGGACTAGCGT-3’ | 204 |
|  | RT6422R | 5’-AAACTGCCATGGAGGTG-3’ |  |
| GRMZM2G040638 | RT0638F | 5’-TGGTGCTGGACAACGCCAAGAT-3’ | 171 |
|  | RT0638R | 5’-CAGTGGTGCAAGCTGATCAAC-3’ |  |
| GRMZM2G172159 | RT2159F | 5’-AAATCTCGCAGGAGGAGGACGTC-3’ | 195 |
|  | RT2159R | 5’-TGAGGCTCTCGATCGAAG-3’ |  |
| AC205413.4_FG001 | RT5413F | 5’-CATCTCCGACAACACACTG-3’ | 198 |
|  | RT5413R | 5’-CTTGACGACTAGTAGTGTGGG-3’ |  |
| GRMZM2G375159 | RT5159F | 5’-TACCAGATCACGGCCGACTAC-3’ | 137 |
|  | RT5159R | 5’-ATGCACGTGGCGATGTAGTCC-3’ |  |
| GRMZM2G126010 | RTActinF | 5’-ATGTTTCCTGGGATTGCCGAT-3’ | 185 |
|  | RTActinR | 5’-CCAGTTTCGTCATACTCTCCCTTG-3’ |  |
| GRMZM2G409726 | RTUbiF | 5’-TCTGGTGCCCTCTCTCCATA-3’ | 167 |
|  | RTUbiR | 5’-TGAGCACAGGCTTTAATTTCA-3’ |  |

Primers used for promoter cloning

| **Promoter** | **Primer** | **Sequences** | **Product length (bp)** |
| --- | --- | --- | --- |
| *p5023* | 5023F | 5’-GACAAGCTTCAGGGTATCCATCACGGC-3’ | 2000 |
|  | 5023R | 5’-TCTAGACTTTTTTCCCCCCC-3’ |  |
| *P8463* | 8463F | 5’-CCCGGGTAGCATGCTTTTGGAGTTA-3’ | 2000 |
|  | 8463R | 5’-CTGCAGGGAAAATATATGAAGC-3’ |  |
| *p6629* | 6629F | 5’-GACCCGGGTCCAACGCCTCGGTCCGACATCTAT-3’ | 1911 |
|  | 6629R | 5’-GACCTGCAGCCTCGACCTCTTTCGTTTGCTTTGG-3’ |  |
| *p8531* | 8531F | 5’-CCCGGGACTGAGGGTATAAGC-3’ | 2000 |
|  | 8531R | 5’-GACTCTAGAAGTCGACGACGATCG-3’ |  |
| *p1534* | 1534F | 5’-GAATTCTGCCTAAGCGTTATG-3’ | 2000 |
|  | 1534R | 5’-TCTAGATATTGCTGCACAGCAGTG-3’ |  |
