# Supplementary material for: Genome-scale mining of root-preferential genes from maize and characterization of their promoter activity
Source: BMC Plant Biol. 2019 Dec 26;19:584. doi: 10.1186/s12870-019-2198-8 (PMC6933907; doi:10.1186/s12870-019-2198-8)
Supplement: Supplementary file 9 — Additional file 9: Figure S1. Relative expression of ubiquitin1, GRMZM2G125023, GRMZM2G308463, GRMZM2G036629, GRMZM2G088531 and GRMZM2G091534. [file 12870_2019_2198_MOESM9_ESM.docx]

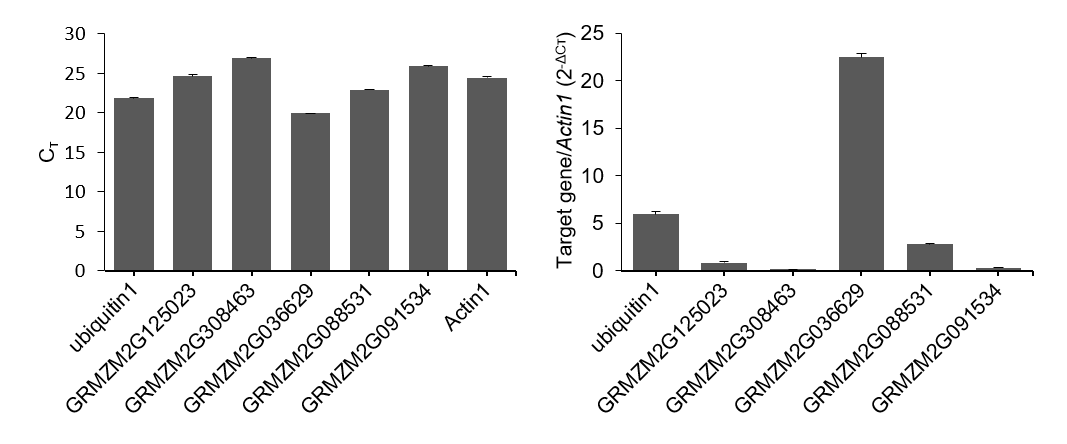


**Additional file 9: Figure S1.** Relative expression of *ubiquitin1*, GRMZM2G125023, GRMZM2G308463, GRMZM2G036629, GRMZM2G088531 and GRMZM2G091534. The roots from flare stage were used for analyses. The abundance of transcripts were compared using C_T_ value, and the relative expression of five root-preferential genes and *ubiquitin1* were normalized to maize *Actin 1* using 2(-ΔC_T_) method. The error bars indicate standard deviations.
